# Sex related differences in nonmotor symptoms of patients with idiopathic blepharospasm

**DOI:** 10.1038/s41598-021-97289-1

**Published:** 2021-09-08

**Authors:** Jing Yang, Lingyu Zhang, Yanbing Hou, Qianqian Wei, Ruwei Ou, Junyu Lin, Wei Song, Bei Cao, Huifang Shang

**Affiliations:** Department of Neurology, West China Hospital, Sichuan University, Chengdu, Sichuan China

**Keywords:** Neurological disorders, Dystonia

## Abstract

Idiopathic blepharospasm shows a female predominance in prevalence, whether there are sex-related differences in distributions of nonmotor symptoms (NMSs) and predictors of quality of life are unknown. Four hundred and twenty-five patients with idiopathic blepharospasm were consecutively recruited, and underwent assessments including dystonia severity, mood disturbances, sleep disturbances, cognition, ocular symptoms, and quality of life. Frequencies and distributions of NMSs, and predictors of quality of life in female and male patients were investigated. NMSs existed in majority of male (94.0%) and female (95.8%) patients. The frequencies of depression, cognition dysfunction, and poor sleep quality were higher in female patients, while the frequency of excessive daytime sleepiness was higher in male patients. More female (79.5%) patients had multiple NMS domains affected than male (70.1%) patients (p = 0.040). Quality of life was associated with depression, anxiety and motor severity for female patients (adjusted R^2^ = 0.367, p < 0.001), while associated with depression, excessive daytime sleepiness and motor severity for male patients (adjusted R^2^ = 0.430, p < 0.001). The highly prevalent coexistence of multiple NMSs found in patients with blepharospasm support that blepharospasm is a network disorder. The sex-related differences in the pattern of NMSs and predictors of quality of life may aid the development of tailored management of blepharospasm.

## Introduction

Idiopathic blepharospasm (BSP) is a focal dystonia characterized by excessive eye blinking and potentially persistent eye closure caused by excessive contraction of orbicularis oculi and adjacent muscles. The pathogenesis is unclear, but it has been suggested to result from the impaired descending control of blink circuits, as a result of dysfunction of cortico-striato-thalamo-cortical circuits. Although BSP is featured by its motor symptoms, growing evidence indicated that BSP patients also present with various nonmotor symptoms (NMSs) including dry eye, photophobia, depression, anxiety, sleep disturbances, and cognitive dysfunction^1^. Studies found NMSs were relevant predictors of health related quality of life (HRQoL) for BSP, and could influence outcome of individual patient after botulinum toxin treatment^2^.

Sex related differences in brain structure and function, hormone levels, genetics, and social attitudes might lead to differences in the clinical expression of neurological diseases. BSP shows a female-to-male preponderance in prevalence, with a reported male-to-female ratio between 1:2 and 1:8^3,4^. However, sex differences in the prevalence and severity of NMSs have been poorly investigated in BSP. Most of previous studies addressing the prevalence and severity of NMSs have focused on individual nonmotor symptom, except a recent study that investigated the coexistence and burden of different NMSs in 60 patients with BSP^5^. Women with BSP have been reported to be more depressed than male patients^2^, but other NMSs haven’t been compared between male and female patients. In addition, no study has explored the effects of multiple NMSs on HRQoL.

In the current study, we investigated motor symptoms and NMSs including depression, anxiety, sleep quality, excessive daytime sleepiness (EDS), cognition and ocular symptoms in a large cohort of idiopathic BSP patients. We aimed to explore differences in the severity and frequency of NMSs and coexistence of multiple NMSs regarding to sex, and to investigate the most important determinants of HRQoL in idiopathic BSP patients.

## Methods

### Participants

A total of 425 BSP patients were consecutively recruited between 2013 December to 2020 January from Department of Neurology, West China Hospital, Sichuan University, and completed clinical assessment. All participants were from neurological outpatient clinic or in-patient ward, and diagnosed by neurologists specialized in movement disorders. Known causes of secondary dystonia were excluded based on the medical and drug histories, neurological examination, laboratory tests and abnormal findings on conventional MRI. Patients receiving botulinum toxin injections were enrolled with an interval of at least 4 months after the last injection, to exclude the potential confounding effects of botulinum toxin treatment.

The ethical committee of West China Hospital of Sichuan University approved the study. Written informed consent was obtained from all participants in accordance with the Declaration of Helsinki.

### Clinical assessment

Participants answered a standardized questionnaire, and the motor symptoms were clinically examined using a standard video protocol^6^. Demographic and clinical characteristics including age, age of onset, disease duration, educational years, family history of dystonia, presence of sensory trick, treatment regimen, and a detailed assessment of motor, NMSs and quality of life, were collected from patients by neurologists specialized in movement disorders through face-to-face interview. The Jankovic rating scale (JRS) was used to evaluate the motor symptoms of BSP patients^7^. Ocular symptoms including photophobia, dryness of eye, pain and red eyes were collected from each patient by administering a questionnaire. The severity of depressive and anxiety symptoms were evaluated by the 24-items Hamilton Depression Rating Scale (HDRS) (a score of 8 or more indicated presence of depression) and the Hamilton Anxiety Rating Scale (HARS) (a score of 7 or more indicated presence of anxiety)^8,9^, respectively. The quality of sleep was measured by the Pittsburg Sleep Quality Index (PSQI), a self-reported questionnaire. PSQI discriminates “poor” from “good” sleepers by evaluating seven aspects of sleep quality including subjective sleep quality, sleep latency, sleep duration, sleep efficiency, sleep disturbances, sleep medication, and daytime dysfunction over the last month. A summed up general score of more than 5 indicates poor quality of sleep^10^. EDS was assessed using the self-administered Epworth Sleepiness Scale (ESS), in which a score ≥ 10 indicates EDS^11^. Cognitive function was evaluated by the Montreal Cognitive Assessment (MoCA). A score of 23 or less indicates cognitive dysfunction based on previous studies in Chinese population^12,13^.

In each subject, we also calculated the overall burden of NMSs, which was defined as the sum of abnormal NMSs and the sum of abnormal NMSs domains. For ocular symptom domain, we used a standardized questionnaire (1 = presence of ocular symptoms; 0 = absence of ocular symptoms); for mood disturbances, we used HARS and HDRS scores (1 = HARS ≥ 7 and/or HDRS ≥ 8, 0 = HARS < 7 and HDRS < 8); for cognition, MoCA total score was used (1 = MoCA ≤ 23, 0 = MoCA > 23); for sleep disturbances, PSQI and ESS scores were applied (1 = PSQI > 5 and/or ESS ≥ 10, 0 = PSQI ≤ 5 and ESS < 10).

The 36-Item Short Form (SF-36) Health Survey was used to evaluate HRQoL for each subject. It consists of 36 items covering 8 domains of physical and mental health: physical functioning (PF), role limitation caused by health problems (RF), bodily pain (BP), perception of general health (GH), Vitality (VT), social functioning (SF), role limitations due to emotional health problems (RE), and mental health (MH). A score from 0 (worst possible health state) to 100 (best possible health state) is generated from each domain^14^.

### Statistical analysis

Demographic and clinical characteristics variables were presented as mean or proportions. Kolmogorov–Smirnov tests were used to examine the normality of variables. Continuous data were presented as mean ± standard deviation, and were compared between female and male patients using Student’s t-test or Mann–Whitney U test or analyses of covariance (ANCOVAs) adjusting for confounding factors. Categorical data were presented as proportions and were compared between female and male patients analyzed by Chi-square exact test. Population data (mean and standard deviation) of eight domains of the SF-36 were taken from a large cross sectional community sample of adult women and men by pooling two age strata, 45–54 and 55–64 years^15^, and compared with female and male BSP patients using a t-test for independent samples.

To explore the relation between motor severity and NMSs, measures including age, sex, education years, disease duration, presence of sensory trick, presence of ocular symptoms, and nonmotor symptom scores (i.e., HARS score, HDRS score, MoCA score, ESS score and PSQI score) were used as independent variables in a multiple regression analyses with backward elimination to determine which were significantly associated with Jankovic total score. Multiple regression analyses with backward elimination were performed to explore the associations between clinical variables and SF-36 total scores in male and female patients, respectively. The dependent variable was SF-36 total score, while the predictors were nonmotor symptom scores (i.e., HARS score, HDRS score, MoCA score, ESS score and PSQI score), motor scores evaluated by Jankovic rating scale, and other clinical variables including age, disease duration, presence of sensory trick, presence of eye disorders. Predictor variables were removed based on F probability > 0.1. Collinearity was examined by variance inflation factor (VIF). Beta values were presented to show the direction of association. R-squared values represent the proportion of total variability explained by independent variables. Partial r-squared value contributed by each significant variable was calculated for the multivariate model. Statistical significance was at p < 0.05. Statistical analysis was performed using BMI SPSS Statistics 22 (SPSS, Inc, Chicago, IL).

## Results

### Demographic and clinical data

Four hundred and twenty five BSP patients were continuously recruited in the current study. Table 1 shows the demographic and clinical data. No significant sex-related differences were found in age, age of onset, disease duration, Jankovic total score, Jankovic frequency score, Jankovic severity score, presence of sensory trick, or proportions of patients receiving botulinum toxin or antidepressants. More female patients were under treatment of trihexyphenidyl, baclofen and benzodiazepines, while more male patients were newly diagnosed and drug naïve.

**Table 1:** Demographic and clinical features of 425 patients with blepharospasm.

	BSP total	Female	Male	P
No. of patients	425	308	117	–
Age	53.23 (10.65)	53.22 (10.09)	53.26 (12.06)	0.975^a^
Onset age	50.27 (10.67)	50.42 (9.77)	49.89 (12.77)	0.682^a^
Disease duration	2.96 (3.68)	2.80 (3.80)	3.37 (4.52)	0.320^b^
Education years	9.80 (3.72)	9.40 (3.64)	10.83 (3.77)	0.001*^a^
Jankovic total score	5.33 (1.50)	5.41 (1.51)	5.13 (1.47)	0.065^b^
Jankovic frequency score	2.64 (0.91)	2.69 (0.91)	2.53 (0.91)	0.106^b^
Jankovic severity score	2.68 (0.76)	2.72 (0.76)	2.58 (0.75)	0.060^b^
Sensory trick (%)	243 (57.2)	173 (56.2%)	70 (59.8)	0.496^c^
HARS	7.16 (6.19)	7.45 (6.18)	6.39 (6.19)	0.054^b^, 1.00^d^
HDRS	9.55 (7.47)	10.16 (7.49)	7.94 (7.20)	0.002*^b^, 0.006*^d^
ESS	5.52 (5.20)	5.13 (5.06)	6.56 (5.43)	0.009*^b^, 0.028*^e^, 0.007*^f^
PSQI	7.57 (4.67)	7.90 (4.64)	6.71 (4.67)	0.009*^b^, 0.006*^e^, 0.172^f^
**MoCA**	24.32 (3.71)	24.01 (3.81)	25.14 (3.25)	0.005*^b^, 0.047*^e^, 0.209^f^
Visuospatial/executive abilities	3.55 (1.25)	3.41 (1.24)	3.92 (1.19)	< 0.001*^b^, 0.007*^e^, 0.024*^f^
Naming	2.41 (0.84)	2.31 (0.89)	2.66 (0.65)	< 0.001*^b^, 0.009*^e^, 0.012*^f^
Attention	5.19 (0.79))	5.16 (0.85)	5.26 (0.63)	0.669^b^, 0.445^e^, 0.820^f^
Language	2.19 (0.89)	2.16 (0.89)	2.27 (0.88)	0.213^b^, 0.791^e^, 0.899^f^
Abstraction	0.99 (0.70)	0.94 (0.69)	1.12 (0.72)	0.019*^a^, 0.377^e^, 0.167^f^
Delayed memory	3.15 (1.50)	3.15 (1.51)	3.16 (1.47)	0.958^a^, 0.717^e^, 0.433^f^
Orientation	5.88 (0.55)	5.85 (0.58)	5.93 (0.45)	0.226^a^, 0.298^e^, 0.357^f^
Drug naïve	217 (51.1%)	139 (45.1%)	78 (66.7%)	< 0.001*^c^
Use of anticholinergics	116 (22.1%)	103 (33.4%)	15 (12.8%)	< 0.001*^c^
Use of Baclofen	98 (23.1%)	95 (30.8%)	5 (4.3%)	< 0.001*^c^
Use of benzodiazepines	97 (22.8%)	90 (29.2%)	9 (7.7%)	< 0.001*^c^
Use of Botulinum toxin	30 (7.1%)	26 (8.4%)	4 (3.4%)	0.071^c^
Use of Antidepressants	20 (4.7%)	11 (3.6%)	9 (7.7%)	0.073^c^

HARS, Hamilton Rating Scale for Anxiety; HDRS, Hamilton Rating Scale for Depression; ESS, Epworth Sleepiness Scale; PSQI, Pittsburg Sleep Quality Index; MoCA, Montreal Cognitive Assessment.

*Significant difference.

^a^Student t-test; ^b^Mann-Whitney U test; ^c^Chi-squared exact test; ^d^ANCOVA with age, education years, disease duration, Jankovic total score, and use of antidepression drug as covariates; ^e^ANCOVA with age, education years, disease duration, Jankovic total score, and use of benzodiazepines, and use of anticholinergics as covariates; ^f^ANCOVA with age, education years, disease duration, Jankovic total score, use of benzodiazepines, use of anticholinergics, HDRS score and HARS score as covariates.

### Sex-related differences in NMSs severities and distributions in BSP patients

The prevalence of NMSs in the total BSP cohort and according to sex is shown in Table 2. In the total BSP patients, the mean (± SD) number of NMSs was 2.9 ± 1.5 (range 0–6), 78.6% of patients complained at least two NMSs and only 4.7% of patients were found to be free of NMSs.

**Table 2:** Prevalence of NMSs and coexistence of NMS domains in 425 BSP patients.

	Total BSP patients, n (%)	Female BSP patients, n (%)	Male BSP patients, n (%)	P
Total number of patients	425	308 (72.5%)	117 (27.5%)	–
Ocular disturbances	303 (71.3%)	225 (74.3%)	78 (66.7%)	0.230
Depression	219 (51.5%)	172 (55.8%)	47 (40.2%)	0.005*
Anxiety	188 (44.2%)	143 (46.4%)	45 (38.5%)	0.156
Cognitive dysfunction	151 (35.5%)	120 (39.0%)	32 (27.4%)	< 0.001*
Excessive daytime somnolence	94 (22.1%)	60 (19.5%)	34 (29.1%)	0.037*
Poor sleep quality	254 (59.8%)	194 (63.0%)	60 (51.3%)	0.035*
**Patients with NMSs**	405 (95.3%)	295 (95.8%)	110 (94.0%)	0.610
Patients with six NMSs	10 (2.4%)	8 (2.6%)	2 (1.7%)	0.734
Patients with five NMSs	59 (13.9%)	46 (14.9%)	13 (11.1%)	0.348
Patients with four NMSs	88 (19.8%)	72 (23.4%)	16 (13.7%)	0.032*
Patients with three NMSs	86 (20.7%)	62 (20.1%)	24 (20.5%)	1.000
Patients with one or two NMSs	162 (38.1%)	107 (34.7%)	55 (47.0%)	0.025*
**Patients with multiple NMS domains**	327 (76.9%)	245 (79.5%)	82 (70.1%)	0.040*
Ocular and mood disturbances	171 (40.2%)	137 (44.5%)	34 (29.1%)	0.004*
Ocular and cognitive disturbances	116 (27.3%)	95 (30.8%)	21 (17.9%)	0.010*
Ocular and sleep disturbances	207 (48.7%)	156 (50.6%)	51 (43.6%)	0.232
Mood and cognitive disturbances	101 (23.8%)	86 (27.9%)	15 (12.8%)	0.001*
Mood and sleep disturbances	195 (45.9%)	149 (48.4%)	46 (39.3%)	0.103
Cognitive and sleep disturbances	108 (25.4%)	86 (27.9%)	21 (17.9%)	0.045*

NMSs, nonmotor symptoms.

*Significant difference.

Compared with male patients, female patients showed significant higher scores of HDRS, but lower scores of ESS, visuospatial/executive abilities and naming subdomains of MoCA, with and without adjustment of confounding factors (Table 1). Female patients had significantly higher PSQI score and lower global MoCA score than male patients, and the differences remain significant after adjusting confounding factors including age, education years, disease duration, Jankovic total score, use of benzodiazepines and anticolinergics, but did not survive after adjusting HDRS score (p = 0.230 for PSQI, p = 0.088 for MoCA) or adjusting both HDRS and HARS scores (p = 0.172 for PSQI, p = 0.209 for MoCA) additionally (Table 1). Female patients complained significantly more frequently than male patients of having depression (p = 0.005) and cognitive dysfunction (p < 0.001), while less prevalent in EDS (p = 0.037) (Table 2). No significant differences were found between female and male patients in the HARS scores, or in the frequency of anxiety or ocular disturbances.

As regards domains, 76.9% patients had more than one domain of NMSs involved, and female patients presented significant higher prevalence of more multiple nonmotor symptom domains affected than male patients (p = 0.040) (Table 2).

### Association between motor severity and NMSs

The multiple regression analysis produced a significant model (R_adj_^2^ = 0.072, p < 0.001), which associated the Jankovic total score with MoCA score (standard. beta = − 0.179, t = − 3.144, p = 0.002), and HARS score (standard. beta = 0.221, t = 3.489, p = 0.001).

### HRQoL and predictors of HRQoL in female and male BSP patients

Both female and male BSP patients scored significantly worse than female and male normal population aged 45–65 years in multiple domains of SF-36 including SF, RF, RE, MH, and GH (Table 3). When compared to male patients, female patients had lower scores in the domain of GH for the HRQoL. No significant differences in SF-36 total score or its other seven domains were found between male and female patients.

**Table 3:** Quality of life between female and male patients with primary blepharospasm.

SF-36	BSP patients				Population aged 45–64			Comparison		
	Total	Female	Male	P^a^	Total	Female	Male	P^b^	P^c^	P^d^
Total scores	515.4 (147.9)	509.2 (147.1)	531.7 (149.5)	0.209	N/A	N/A	N/A	N/A	N/A	N/A
PF	83.0 (19.9)	82.2 (19.5)	85.1 (20.8)	0.068	82.4 (20.8)	80.5 (21.3)	84.3 (20.1)	0.575	0.168	0.679
SF	70.6 (23.5)	69.7 (24.0)	72.8 (22.0)	0.316	81.6 (33.5)	80.5 (21.3)	84.3 (20.1)	< 0.001*	< 0.001*	< 0.001*
RF	36.9 (43.2)	37.1 (43.4)	36.4 (43.0)	0.956	81.6 (33.5)	79.8 (34.4)	83.6 (32.4)	< 0.001*	< 0.001*	< 0.001*
RE	54.3 (45.2)	54.1 (45.3)	54.9 (45.1)	0.957	83.7 (31.6)	81.9 (33.1)	85.7 (29.7)	< 0.001*	< 0.001*	< 0.001*
MH	63.8 (21.1)	62.7 (21.0)	66.9 (21.0)	0.058	75.2 (17.8)	73.7 (18.3)	76.9 (17.0)	< 0.001*	< 0.001*	< 0.001*
VT	66.7 (20.8)	65.9 (20.9)	68.9 (20.3)	0.282	61.0 (20.5)	59.2 (20.8)	62.9 (20.1)	< 0.001*	< 0.001*	0.002*
BP	87.8 (17.4)	87.2 (17.9)	89.3 (16.1)	0.298	78.3 (23.4)	76.3 (23.6)	80.4 (22.9)	< 0.001*	< 0.001*	< 0.001*
GH	52.6 (23.7)	50.8 (23.2)	57.4 (24.2)	0.013*	70.5 (21.2)	70.9 (21.0)	70.2 (21.5)	< 0.001*	< 0.001*	< 0.001*

PF, physical functioning; RF, role limitation caused by health problems; BP, bodily pain; GH, perception of general health; VT, Vitality; SF, social functioning; RE, role limitations due to emotional health problems; MH, mental health.

*Significant difference; P^a^ female BSP patients vs. male BSP patients; P^b^ female BSP patients vs. female population aged 45 to 65 years old; P^c^ male BSP patients vs. male population aged 45 to 65 years old; P^d^ total BSP patients vs. the population aged 45–65 years old.

Backward stepwise regression analysis produced a significant model, which associated SF-36 total score with HDRS score, HARS score and Jankovic total score for female BSP patients, and the HDRS had the highest partial r-squared followed by Jankovic total score among the significant independent variables. In a separate model predicting SF-36 score for male patients was significant with predictors including HDRS, ESS and Jankovic total score; and the HDRS had the highest partial r-squared followed by ESS, suggesting that HDRS and ESS were the most important determinants for HRQoL for male patients (Table 4).

**Table 4:** Results of backward stepwise linear regression analyses for predictors in HRQoL with the clinical variables in total BSP patient group, and female and male patient subgroups.

Patient group	R_adj_^2^	F	P	Predictor(s)	Standard beta	t	p	VIF	Partial r-squared
Total sample (n = 425)	0.390	50.845	< 0.001*	HDRS	− 0.410	− 5.475	< 0.001*	2.862	0.0888
				HARS	− 0.188	− 2.503	0.013*	2.885	0.0199
				JRS total score	− 0.165	− 3.676	< 0.001*	1.026	0.0420
Female group (n = 308)	0.367	45.368	< 0.001*	HDRS	− 0.405	− 4.709	< 0.001*	2.687	0.0888
				HARS	− 0.197	− 2.287	0.023*	2.706	0.0225
				JRS total score	− 0.149	− 2.804	0.005*	1.022	0.0335
Male group (n = 117)	0.430	16.265	< 0.001*	HDRS	− 0.540	− 6.293	< 0.001*	1.047	0.3399
				ESS	− 0.163	− 1.918	0.049*	1.027	0.0458
				JRS total score	− 0.165	− 1.917	0.059	1.056	0.0454

HDRS, Hamilton Rating Scale for Depression; HARS, Hamilton Rating Scale for Anxiety; JRS total score, Jankovic rating total score; ESS, Epworth Sleepiness Scale.

*Significant difference.

## Discussion

This is the first study to explore differences in the distributions of NMSs and predictors of HRQoL in idiopathic BSP patients between different sexes. In a large cohort of BSP patients, coexistence of multiple NMSs were highly prevalent in all patients, and a higher prevalence of depression, poor sleep quality, and cognitive dysfunction were found in female patients, while EDS was more prevalent in male patients. HRQoL was significantly correlated with motor and nonmotor symptoms in BSP patients. Subgroup analysis revealed that motor severity, depression and anxiety were the determinants of HRQoL for female patients, whereas depression and EDS were the significant predictors for male patients.

Previous studies indicated that patients with dystonia had higher than expected rates of depression and anxiety, with a reported prevalence ranging from 21 to 71%^16^. However, most of the studies were based on a sample of patients with mixed type of dystonia, and only few focused on BSP specifically and none has explored the sex effect on the prevalence. The current study with a sample of 425 BSP patients found that 44.2% of patients had anxiety and 51.5% of patients complained depressive symptoms. This prevalence falls into the most frequently reported range in studies with mixed type of dystonia, but it was higher than that reported in a prior study, which reported 37.2% of one study including 89 BSP patients^2^. We also found female BSP patients being significantly more depressed than male patients, even after adjusting for the motor severity, which was consistent with the previous study^2^. Although the reason for a preponderance of depression in women for BSP is unknown, the fact that this sex difference in depression was consistently found in community-based epidemiological studies and in major depression disorder incidence across cultures suggests that there would be biological differences apart from culture, diet, education, and numerous social and economic factors that place women at increased risk^17^. Evidence indicates that there were sex differences in depression-related transcriptional patterns, changes in neuroanatomy and neuroplasticity, and immune signatures, leading to greater susceptibility to depression in female^18^. Recent neuroimaging studies in blepharospasm revealed that structural and functional abnormalities in limbic system^19,20^, which involved in mood regulation and showed sex differences in morphometry^21^, might provide the pathologic substrates for the high prevalence of mood disorders and the preponderance of depression in female for BSP.

By evaluating the sleep quality using PSQI, 59.8% of BSP patients reported poor sleep quality. The prevalence is similar to our previous study with a prevalence of 55% in 60 BSP patients and lower than another prior study with 75% of 52 BSP patients^22,23^; both studies found the prevalence of poor sleep quality in BSP was significantly higher than those reported in healthy controls. The etiology of poor sleep quality in BSP is unknown, but the primary effects of movement of dystonia and secondary effects of depression and drugs may play a role. In addition, the observed associations between poor sleep quality and BSP may be alternatively linked to the abnormal enhanced excitability of brainstem circuits^24^, which need further studies to elucidate. In an early polysomnographic study of ten patients with BSP and oromandibular dystonia, abnormal movement progressively decreased but did not disappear during sleep, and impaired sleep efficiency and decreased REM sleep were reported to correlate with the severity of dystonia especially in patients with lower craniocervical muscles involved^25^. However, no changes in sleep architecture and organization were found in another study on focal and generalized dystonia^26^. Our regression analysis showed that motor severity was not associated with sleep quality, which was consistent with a previous study on BSP^23^. Meanwhile, a sex effect on sleep quality was found in BSP patients, and this effect was not related to motor severity or administration of benzodiazepines or anticholinergics, but appeared to be because of a confounding effect of depression. Whether BSP patients have altered sleep structure and its relation with motor abnormality need further study.

ESS was used to evaluate the daytime sleepiness, and 22.1% of all BSP patients had EDS. ESS score was found not related to motor severity, which was in consistent with previous findings of cervical dystonia^27^. In contrast to more prevalent of poor sleep quality in female patients, male patients reported higher scores of ESS and more frequent of EDS. The association between EDS and male was reported in general adult population^28,29^ and in patients with Parkinson’s disease^30^. The underlying mechanism of EDS in BSP differs between sexes was unclear. Factors such as depression and use of anti-dystonia medication may contribute to such discrepancy. However, the sex effect on EDS in BSP survived with adjustment of disease duration, motor severity, administration of benzodiazepine and anticholinergic medication, and also depression and anxiety scores, this indicated that the sex effect on EDS in BSP is not associated with motor severity, disease duration, drug administration, anxiety or depression. Cumulative evidence suggested that sex differences existed in circadian timing system in the hypothalamic–pituitary–gonadal axis, the hypothalamicadrenal-pituitary axis, and sleep-arousal systems^31^, whether these sex differentiated circadian timing systems was associated with pathogenesis of BSP needs further study.

Prior studies focused on certain domains of cognition were of small sample sizes and found that patients with primary focal dystonia had statistically significant deficits in attentional, executive, memory or visuospatial function^32,33^. Using MoCA, we found 35.5% of BSP patients had cognitive dysfunction, which was similar with the finding (32.3%) of our previous study using Addenbrooke’s Cognitive Examination-Revised (ACE-R)^34^. Although anti-dystonia medications such as anticholinergic medication and benzodiazepine may affect the cognitive performance, the subgroup analysis of the drug naïve patients found that 39.6% of patients had cognitive dysfunction. It supported that cognitive dysfunction observed in our patients with BSP was not caused by the anti-dystonia medications. The weak association between MoCA score and motor severity suggested that the cognitive deficits were not secondary to the motor disabilities, which was consistent with our previous studies^22,34^. A sex effect on cognitive deficits of selective domains was found in BSP patients, as female patients reported poorer cognitive performance in visuospatial/executive abilities and naming domains than male patients after adjusting confounding factors including age, education years, disease duration, motor severity, depression, anxiety and anti-dystonia medications. Gender differences in cognitive function were identified in adulthood and ageing. Our finding was consistent with previous studies that men outperformed women on spatial tasks^35^, and that men had higher naming scores on MoCA than women in normal aging Chinese population^36^. Besides differences in genetics, environmental exposure, and sexual hormones between female and male patients, neurobiological sex differences in brain regions implicated in cognition exist at multiple levels, from gross neuroanatomy to circuit properties to molecular mechanisms under them, may contribute to these sex differences in cognition^35,37^. Whether these gender differences in cognition performance are associated with pathology of BSP needs further study.

A high prevalence (78.6%) of coexistence of multiple NMSs was found in BSP patients, and more than half of patients complained at least three NMSs. The frequency of coexistence of multiple NMSs is similar with that (71.6%) reported in a previous study including 60 patients with BSP^5^. Although the pathophysiology and neuroanatomy of BSP have not been fully elucidated, the wide spread involvement of cortical and subcortical regions revealed by neuroimaging studies indicated that it may be a network disorder and may provide the basis for the development of various NMSs^38,39^. The coexistence of multiple NMSs emphasizes the need for evaluating and managing both motor and nonmotor manifestations in BSP. Further studies are necessary to explore the longitudinal evolution of nonmotor symptoms and their response to treatments, and whether they have influence on the prognosis of dystonia.

When compared to the population aged 45–65 years, both female and male patients with BSP were scored significantly lower in the majority domains of SF-36. Interesting, although sex-related differences were shown in the prevalence and severity of NMSs, there were no significant differences in terms of all domains of SF-36 except for GH. This may indicate NMSs have different influences on HRQoL for female and male patients. Among all the clinical symptoms including nonmotor and motor symptoms, depression was found to be the most important factor affecting HRQoL in BSP patients. Depression and motor severity were the determinants of HRQoL for both female and male patients. The other determinant of HRQoL for female patients was anxiety while for male patients was EDS, which may due to the ESS scores were significantly higher in male than female. These findings highlight the importance of identifying and treating both motor and nonmotor manifestations, and should pay attention to sex disparities when devising patient management strategies.

Several caveats need to be considered when interpreting our results. Firstly, this is not a population-based study but involves patients recruited from a single health center. Second, we did not conduct the structured clinical interviews using the Diagnostic and Statistical Manual of Mental Disorders to establish the definite diagnosis of depression or anxiety. However, HDRS and HARS are available scales to screen for symptoms of depression and anxiety, respectively. Third, we did not use comprehensive neuropsychological batteries to test cognitive function and mood conditions in more detail. Lastly, the lack of a group of healthy controls is an important limitation. However, in current study the nonmotor symptoms were assessed with scales that were widely used in dystonia and other movement disorders, and the cutoff values of these scales used in this study were widely recommended and applied in clinical research, it may facilitate comparisons across studies.

## Conclusions

In conclusion, this study with a large sample size indicated the existence of sex-related differences in the patterns of NMSs and their effects on HRQoL in idiopathic blepharospam patients. Such differences in the prevalence and severity of NMSs and their roles in HRQoL could open new insight in the management of bleparospasm and promote the understanding of the disease.

## Data Availability

The data that support the findings of this study are available on request from the corresponding author.
